# Time-differentiated target temperature management after out-of-hospital cardiac arrest: a multicentre, randomised, parallel-group, assessor-blinded clinical trial (the TTH48 trial): study protocol for a randomised controlled trial

**DOI:** 10.1186/s13063-016-1338-9

**Published:** 2016-05-04

**Authors:** Hans Kirkegaard, Bodil S Rasmussen, Inge de Haas, Jørgen Feldbæk Nielsen, Susanne Ilkjær, Anne Kaltoft, Anni Nørregaard Jeppesen, Anders Grejs, Christophe Henri Valdemar Duez, Alf Inge Larsen, Ville Pettilä, Valdo Toome, Urmet Arus, Fabio Silvio Taccone, Christian Storm, Markus B. Skrifvars, Eldar Søreide

**Affiliations:** Department of Anaesthesiology and Intensive Care Medicine, Aarhus University Hospital and Aarhus University, Aarhus, Denmark; Department of Anaesthesiology and Intensive Care Medicine, Aalborg University Hospital, Aalborg, Denmark; Hammel Neurorehabilitation Centre and University Research Clinic, Aarhus University, Hammel, Denmark; Department of Anaesthesiology and Intensive Care Medicine, Aarhus University Hospital, Aarhus, Denmark; Department of Cardiology, Aarhus University Hospital, Aarhus, Denmark; Department of Cardiology, Stavanger University Hospital, Stavanger, Norway; Department of Clinical Science, University of Bergen, Bergen, Norway; Division of Intensive Care, Department of Anesthesiology and Intensive Care Medicine, Helsinki University Hospital and Helsinki University, Helsinki, Finland; Intensive Care, Inselspital, Bern University Hospital, Bern, Switzerland; Department of Anesthesiology, Intensive Care and Emergency Medicine, North Estonia Medical Centre, Tallinn, Estonia; Department of Intensive Cardiac Care, North Estonia Medical Centre, Tallinn, Estonia; Department of Intensive Care, Hôpital Erasme, Université Libre de Bruxelles (ULB), Brussels, Belgium; Department of Internal Medicine, Nephrology and Intensive Care, Charité-Universitätsmedizin Berlin, Berlin, Germany; Department of Anaesthesiology and Intensive Care, Stavanger University Hospital, Stavanger, Norway; Department of Clinical Medicine, University of Bergen, Bergen, Norway

**Keywords:** Out-of-hospital cardiac arrest, Target temperature management, Mild therapeutic hypothermia, Prolonged target temperature management

## Abstract

**Background:**

The application of therapeutic hypothermia (TH) for 12 to 24 hours following out-of-hospital cardiac arrest (OHCA) has been associated with decreased mortality and improved neurological function. However, the optimal duration of cooling is not known. We aimed to investigate whether targeted temperature management (TTM) at 33 ± 1 °C for 48 hours compared to 24 hours results in a better long-term neurological outcome.

**Methods:**

The TTH48 trial is an investigator-initiated pragmatic international trial in which patients resuscitated from OHCA are randomised to TTM at 33 ± 1 °C for either 24 or 48 hours. Inclusion criteria are: age older than 17 and below 80 years; presumed cardiac origin of arrest; and Glasgow Coma Score (GCS) <8, on admission. The primary outcome is neurological outcome at 6 months using the Cerebral Performance Category score (CPC) by an assessor blinded to treatment allocation and dichotomised to good (CPC 1–2) or poor (CPC 3–5) outcome. Secondary outcomes are: 6-month mortality, incidence of infection, bleeding and organ failure and CPC at hospital discharge, at day 28 and at day 90 following OHCA. Assuming that 50 % of the patients treated for 24 hours will have a poor outcome at 6 months, a study including 350 patients (175/arm) will have 80 % power (with a significance level of 5 %) to detect an absolute 15 % difference in primary outcome between treatment groups. A safety interim analysis was performed after the inclusion of 175 patients.

**Discussion:**

This is the first randomised trial to investigate the effect of the duration of TTM at 33 ± 1 °C in adult OHCA patients. We anticipate that the results of this trial will add significant knowledge regarding the management of cooling procedures in OHCA patients.

**Trial registration:**

NCT01689077

## Background

Out-of-hospital cardiac arrest (OHCA) is a major health problem associated with significant morbidity and mortality. Post-anoxic brain damage remains the leading cause of death in those patients surviving admission to the intensive care unit (ICU) [1]. Extended brain injury is caused by a period of ischaemia followed by the reperfusion phase (the so-called ‘ischaemia-reperfusion injury’) [2], which will result in the activation of several pathophysiological pathways, including the release of glutamate, intracellular calcium accumulation, production of reactive oxygen species and an increased inflammatory response. These phenomena will induce neuronal and astroglial cell death with irreversible loss of cerebral functions [3–5].

Previous animal studies have shown beneficial effects on survival and neurological recovery with mild induced hypothermia, also called therapeutic hypothermia (TH), after cardiac arrest [6, 7]. In 2002, two randomised clinical studies demonstrated that TH, with cooling of the patient to 32–34 °C, decreased mortality and improved neurological function in unconscious OHCA survivors [8, 9] This led to a progressive and widespread implementation of TH as a standard treatment in comatose OHCA patients, and its use was recommended by international resuscitation guidelines for all comatose post-anoxic survivors, regardless of the location of arrest and the initial rhythm [10]. The exact neuroprotective mechanisms of TH on the ischaemia-reperfusion injury have not been fully elucidated, but they involve, among other processes, a reduction of brain metabolism, the attenuation of glutamate and dopamine release and the inhibition of apoptosis [11–14].

Based on international consensus, the term therapeutic hypothermia has now been replaced with the term targeted temperature management (TTM) [15]. One unresolved issue concerning the use of TTM after cardiac arrest is the optimal duration of cooling. In the large randomised controlled trials (RCTs) [6, 7, 16], cardiac arrest (CA) survivors were cooled for between 12 and 24 hours. However, experimental data suggest an additional cerebral benefit from prolonged cooling (for 48 hours or longer). In a CA swine model, Suh et al. showed that the application of TTM for 48 hours resulted in a higher attenuation of neuronal apoptosis when compared to 24-hour treatment [17]. In a rat CA model, histological assessment of neuronal survival revealed a greater neuroprotection with prolonged (48 hours) TTM compared to 24-hour intervention [18]. In adult patients, there are no randomised trials investigating the effect of prolonged TTM. A small number of observational studies showed no difference in mortality or poor neurological outcome [19, 20], but prolonged TTM of up to 72 hours was feasible and safe and associated with a less pronounced burden of inflammatory response during rewarming in patients suffering from post-anoxic brain injury [21]. Considering the scientific equipoise between 24-hour and a longer duration of cooling in the CA setting, we designed a trial aiming to test whether 48 hours of TTM at 33 ± 1 °C is superior to 24-hour therapy in unconscious OHCA survivors. In addition, we aimed to assess the safety of prolonged cooling, in particular in regard to the occurrence of adverse reactions and prolongation of ICU care. This manuscript is prepared according to the SPIRIT guidelines [22].

### Objective

The aim of this study is to investigate the effects of a prolonged duration (48 hours) of TTM at 33 ± 1 °C (TTM33) in unconscious OHCA patients compared to standard therapy (24 hours).

### Hypothesis

We hypothesise that cooling for 48 hours results in a higher proportion of CA patients with good neurological recovery (assessed by the Cerebral Performance Category score (CPC) [23, 24], Table 1.) at 6 months compared to 24 hours therapy. CPC 1–2 is defined as a good neurological outcome and CPC 3–5 as a poor neurological outcome.

**Table 1:** Cerebral Performance Categories Score

CPC1. Good cerebral performance
Conscious. Alert, able to work and lead a normal life. May have minor psychological or neurological deficits (mild dysphasia, non-incapacitating hemiparesis, or minor cranial nerve abnormalities)
CPC 2. Moderate cerebral disability
Conscious. Sufficient cerebral function for part-time work in a sheltered environment or independent activities of daily life (dressing, traveling by public transportation, and preparing food). May have hemiplegia, seizures, ataxia, dysarthria, dysphasia, or permanent memory or mental changes
CPC 3. Severe cerebral disability
Conscious. Dependent on others for daily support because of impaired brain function (in an institution or at home with exceptional family effort). At least limited cognition. Includes a wide range of cerebral abnormalities from ambulatory with severe memory disturbance or dementia precluding independent existence to paralytic and able to communicate only with eyes, as in the locked-in syndrome
CPC 4. Coma, Vegetative state
Not conscious. Unaware of surroundings, no cognition. No verbal or psychological interactions with environment
CPC 5. Death
Certified brain dead or dead by traditional criteria

### Trial design

The TTH48 study is a pragmatic, randomised, prospective, assessor-blinded, multicentre trial with two arms: a control arm (24 hours’ TTM33) and an intervention arm (48 hours’ TTM33).

## Methods: participants, intervention and outcomes

### Study setting

The TTH48 study is currently ongoing in eight ICUs at seven hospitals in six European countries. The participating institutions are listed at the homepage of the trial (www.tth48.com), and the trial has been registered at www.clinicaltrials.gov NCT01689077).

### Eligibility criteria

All unconscious OHCA patients entering the ICUs are screened for inclusion criteria. Informed consent and randomisation to one of the two interventional arms should occur within the first 23 hours after a body temperature of 34 °C or colder is reached. Informed consent will be obtained from all participants.

Patients must meet all the following inclusion criteria:

1. Age over 17 years and below 80 years
2. OHCA with a presumed cardiac origin
3. Sustained spontaneous circulation after resuscitation (no need for cardiac compressions during 20 min and clinical signs of circulation)
4. Glasgow Coma Score (GCS) [25] <8 on admission

The inclusion criteria include, as opposed to those in previous RCTs [8, 9], both patients with shockable and non-shockable rhythms.

Patients fulfilling one of the following criteria are excluded:

1. Estimated time interval from collapse to return of spontaneous circulation over 60 min
2. CA with presumed non-cardiac cause (e.g. trauma, aorta dissection, intracerebral disease, massive bleeding, hanging or hypoxemia)
3. In-hospital cardiac arrest
4. Terminal disease or ‘not-to-be reanimated’ orders
5. Severe coagulopathy (anticoagulant therapy, including thrombolysis, is not an exclusion criteria)
6. Unwitnessed OHCA with asystole as first rhythm
7. Time from cardiac arrest to initiation of cooling longer than 240 min
8. Pregnancy
9. Previous neurological disease with cognitive impairment
10. Persistent cardiogenic shock, systolic blood pressure <80 mmHg despite vasoactive treatment and/or aortic balloon pump intervention
11. Suspected or confirmed acute intracerebral bleeding
12. Suspected or confirmed acute stroke
13. Acute coronary artery bypass surgery
14. Lack of consent from next of kin or general practitioner/medical officer of health or from the patient if they wake up and is capable. This criterion should be used in accordance with local ethical requirements

### Interventions

The use of TTM should be initiated as quickly as possible, if possible within 60 min after the return of spontaneous circulation (ROSC). There are no restrictions on the methods to be used to induce TTM. The maximal amount of cold fluids (4 °C) to be given in these patients is 30 ml/kg, particularly for TTM induction. The target temperature is set at 33 ± 1 °C and measured in the bladder, rectum, nasopharynx or blood, according to local practices. The achievement of the target temperature is considered at a body temperature of 34 °C or colder. At the end of the cooling period, rewarming is progressively achieved at 0.5 °C/h until a body temperature of 37 °C is reached. At that moment, sedative/analgesic agents are stopped and neurological assessment (including clinical examination and additional prognostic tools) of the patient is conducted daily. Patients who eventually wake up and are extubated are observed for at least 24 hours before ICU discharge.

#### Withdrawal of active treatment

All active treatment is continued until 72 hours after normothermia has been reached. Exceptions to this may include patients who develop clinical signs of brain death or refractory shock with multiple organ dysfunctions. In addition, patients with previously unrecognised terminal cancer at the initiation of treatment may undergo a limitations on life-sustaining therapies procedure (LLSTP) in the event that further medical treatment is considered unethical. These decisions are made by the attending physician in a multidisciplinary fashion (e.g. including neurologists and neurophysiologists whenever possible), independently from the research team. Patients who remain comatose 72 hours after normothermia are assessed with a combination of daily neurological examination (including at least the evaluation of motor response to noxious stimuli and pupillary/corneal reflexes), continuous or repeated intermittent electroencephalogram (EEG) and somatosensory evoked potentials (SSEPs). The decision to initiate LLSTP is based on agreement among the participating centres [26].

#### Interruption of hypothermic procedures

There is a possibility that the cooling treatment may be associated with uncontrolled bleeding, life-threatening arrhythmias or refractory low cardiac output (e.g. despite maximal inotropic/vasopressor therapy). If one of these complications should occur, the cooling treatment can be stopped according to the decision of the attending physicians and medical team, independently from the research team. The excluded patient will then be kept at 36 °C throughout the intervention period and considered in the treatment arm of randomisation for final outcome according to the intention-to-treat analysis.

#### Concomitant therapies

Concomitant medication will follow local guidelines and may vary between study sites. Sedation is managed with propofol or midazolam and analgesia with remifentanyl or fentanyl. The use of neuromuscular blocking agents (cisatracurium or rocuronium) is recommended until the target temperature is achieved, and it should only be continued thereafter if needed (e.g. if shivering occurs). Convulsions should be aggressively treated with sedative agents and anti-epileptic drugs according to standard operating procedures. Mean arterial pressure (MAP) should be targeted above 60–65 mmHg. If circulatory support is needed, the choice of the inotropic/vasopressor agent is dependent on the attending physician. Blood glucose should be maintained between 6 and 10 mmol/l using glucose or insulin infusion administered intravenously, if required.

### Outcome

The primary outcome of the study is the proportion of patients with good neurological outcome (CPC 1–2) at 6 months after CA (Table 1). The CPC assessment will be scored during a semi-structured telephone interview or person-to-person interview performed by an assessor blinded to the treatment allocation.

**Fig. 1:**
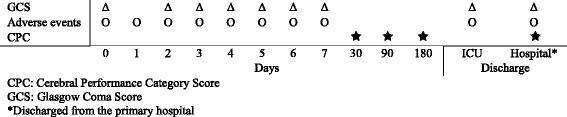
Outcome. Outcome assessment over the study period

Secondary outcomes: (Fig. 1)

1. CPC at hospital discharge and at 30 and 90 days
2. GCS on day 4 and at hospital discharge
3. Mortality (CPC 5 at 6 months)
4. Adverse reactions (Table 2)

**Table 2:** Adverse events. Adverse events and definitions, reported from day 1 to discharge from the primary hospital

Adverse events	Definitions	
Cerebral	Pupils	Reacting to light, dilated (size), dilation difference between eyes
	Seizure	Involuntary contractions or series of contractions of the voluntary muscles
	Myoclonus	Short lasting involuntary contractions of one or several muscles
	Myoclonus state	Continuous myoclonus
	Convulsive state	Continuous seizures or continuous seizure pattern on EEG
Circulation	Hypotension	Mild: MAP >60 mmHg with one inotropic agent and volume infusion
		Moderate: MAP >60 mmHg with full treatment
		Severe: MAP 50–60 mmHg despite full treatment
		Circulatory failure: MAP <50 mmHg for more than 10 min despite full treatment
	Need for pacing	
	Resuscitation	
Arrhythmias	Mild	Arrhythmias that do not demand treatment
	Moderate	Stable haemodynamics (MAP >60 mmHg) with treatment
	Severe	Pulseless VT/VF or unstable haemodynamics despite treatment
Gastrointestinal	Mild	Aspiration, can partly take enteral nutrition
	Moderate	Aspiration more than 400 ml, cannot take any enteral nutrition
	Severe	Ileus, bleeding gastric ulcer, need for explorative laparotomy or others
Urological	Continuous or intermittent replacement therapy	
Infectious/inflammatory	Pneumonia	New or progressing infiltrations on thoracic X-ray
		Fever (not during hypothermia treatment)
		Leucocytosis
		Purulent tracheobronchial secretion
	SIRS^a^	At least 2 of 4 SIRS criteria present
	Sepsis	Sepsis, SIRS caused by an infection
	Severe sepsis	Sepsis associated with organ dysfunction
	Septic shock	Sepsis with hypotension^b^
Bleeding and transfusion	Bleeding	Mild: no transfusion needed
		Moderate: up to two RBC units/24 hours.
		Severe: more than two RBC units/24 hours
		Critical bleeding in organs: intracranial, intrathecal, intraocular or pericardial
		Other bleeding: retroperitoneal, thorax or solid organs
	Transfusion	Number of transfusions

*EEG* electroencephalogram, *MAP* mean arterial pressure, *VF* ventricular fibrillation, *VT* ventricular tachycardia

^a^SIRS: temperature >38 °C or <36 °C, pulse rate >90 beats/min, respiratory rate >20 breath/min or PaCO_2_ < 4.3 kPa or need for mechanical ventilation, leucocytes >12,000 cells/mm^3^, or <4000 cells/mm^3^, or >10 % immature cells

RBC: red blood cell

^b^systolic blood pressure <90 mmHg or a reduction >40 mmHg from baseline and perfusions abnormalities or need for vasoactive drugs despite adequate volume treatment in the absence of other reasons for hypotension
5. Progression of GCS from days 1 to 7 in the ICU and at ICU discharge

### Sample size

Assuming a proportion of patients with good cerebral outcome (CPC 1–2) following a 24-hour TTM treatment of 50 % [8, 9] and an expected absolute 15 % difference in the good outcome rate between the groups (two-sided), 169 patients in each group (*n* = 338) would be required to have a study power of 80 % and a 5 % significance. However, this power calculation does not take into consideration possible missing outcome data, and thus the total number of patients in the study is increased to 350.

### Recruitment

**Fig. 2:**
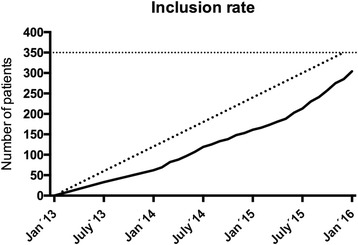
Inclusion rate. Inclusion rate up to the first 304 patients (1 February 2016). Allocation of the last of the 350 patients is anticipated to be late May 2016. The solid line represents included patients; the broken line represents the expected inclusion rate

Patient screening and inclusion started on 1 February 2013. Recruitment is ongoing and, as of 1 February 2016, 304 patients have been included and randomised into one of two groups. Allocation of the last of the 350 patients is anticipated to be late May 2016 (Fig. 2).

## Methods: assignment of intervention

### Allocation

The centres will have access to a web-based randomisation procedure (Trial Partner, Institute for Clinical Medicine, Aarhus University, Denmark). Each patient will receive his or her own randomisation number. Randomisation is balanced at a 1:1 ratio and will take place in blocks of random size, stratified according to the study site, age (above and below 60 years) and initial rhythm (shockable versus non-shockable rhythm).

### Sequence generation

The randomisation process was created by a data manager not involved in patient recruitment. The screening and randomisation procedure was built in a web-based chart record form, sampling data from the study (e-CRF).

### Blinding

The registration and measurement of 6-month CPC are done by research staff members who are blinded to the study treatment arm. Registration of adverse events and GCS scores, while the patient is in the ICU, is performed by the treating physician team and research staff, who are not blinded to the treatment group since the attending staff cannot be blinded to the intervention. Information about the intervention group is limited to those treating the patients. Relatives and patients are not blinded to the intervention.

## Methods: data collection, management and analyses

### Data collection and management (Fig. 3)

**Fig. 3:**
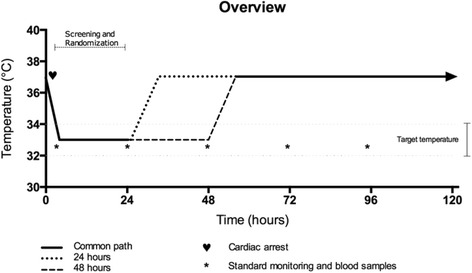
Trial overview. Trial overview according to the two interventional arms

Data collection follows the Utstein style [27]. The study data are entered online in an Internet-based database and collected from hospital records, ambulance records and national databases. It is, therefore, assumed that the research staff have access to these data sources, including the electronic medical records (EMR). Each centre is responsible for: (1) screening and registration of all comatose OHCA patients admitted to the ICU, (2) randomisation, (3) treatment, (4) collection and entry of data according to the study protocol and the e-CRF, and (5) obtaining informed consent. Any violation/deviation of the protocol must be recorded in the e-CRF. The trial data comprise e-CRF data extracted by the data manager in co-operation with the principal investigator of the study. The data are made accessible on an international open database (ClinicalTrials.gov) and kept for 15 years after completion of data collection for the basic study and then destroyed.

### Statistical methods

The primary outcome measure CPC will be divided into two groups: group 1 = patients with a good neurological outcome (CPC 1–2); group 2 = patients with a poor neurological outcome (CPC 3–5). Categorical data (group 1 and group 2 outcomes) will be compared using a chi-square test. Continuous parameters/data will be assessed for normality and compared using the Student’s *t* test or Mann-Whitney *U* test, accordingly. A *p* value <0.05 is considered statistically significant. Secondary outcome measures will be compared between groups using non-parametric or parametric tests. Descriptive statistics will be calculated for the two groups. A predefined plan for further data analyses will be prepared in collaboration with a biostatistician. Data will be analysed according to the ‘intention-to-treat’ principle. The report will encompass the data recommended by the Consolidated Standards of Reporting Trials (CONSORT) Statement for non-pharmacological treatment interventions.

## Methods: monitoring

### Data monitoring

The trial is followed by a Data and Safety Monitoring Committee (DSMC). When 175 of the 350 patients have been allocated, a safety interim analysis will be performed comparing serious adverse reactions (SAR), including death, between the two arms of the trial. Blinded data will be analysed by an independent statistician and provided to the DSMC. Based on the analyses of SAR, the DMSC will use the Haybittle-Peto statistical approach to guide its recommendations regarding early termination or continuation of the trial. If appropriate, the DSMC may receive un-blinded data. The Trial Steering Committee, comprising up to two members from each centre and the principal investigator as the chairman of the committee, will make the final decision regarding the continuation or discontinuation of the trial. Further interim analyses will be based on the recommendations of the DSMC.

The safety analyses have been performed and the DSMC had no comments, but recommended a safety interim analysis comparing the main outcome parameter CPC at 6 months for the first 175 patients.

### Harm

Each year throughout the trial, the principal investigator must submit a list to the ethical committee in the Central Denmark Region of all serious expected/unexpected adverse events that have occurred during the period. This information must be accompanied by an assessment of the subjects’ safety.

## Ethics and dissemination

### Ethical approval

The protocol was approved by:

The Ethics Committee of Central Denmark Region on 2 January 2011 (journal number 20110022).

The Regional Ethics Committee of Western Norway on 9 October 2013 (ref 2013/1486).

The Ethical Committee of Helsinki and Uusimaa Hospital District on 21 October 2014 (§157).

The Tallinn Ethical Committee, Estonia on 10 March 2015 (approval number. 943).

The Ethics Committee of the Hôpital Erasme, Brussels, Belgium on 9 March 2015 (021/406).

The Ethics Committee of the Charité-Universitätsmedizin, Berlin on 2 August 2015 (EA2/080/15).

The trial is performed according to the current version of the Helsinki Declaration (2013). The study is conducted in accordance with the present protocol, the ICH-GCP guidelines and the regulatory requirements that apply to the study.

### Withdrawal of consent

Patients who wake up will be informed verbally and in writing about the study they are participating in and asked to continue in the study. If a patient refuses, he or she will be asked for permission to use the data collected before the withdrawal of his or her consent and for permission to collect primary data, i.e. CPC after 6 months.

### Confidentiality

The trial protocol is approved by The Danish Data Protection Agency.

### Dissemination policy

The results of the study will be published in a peer-reviewed international medical journal.

## Discussion

Current resuscitation guidelines recommend TTM treatment aiming at a target temperature of between 32 and 36 °C for at least 24 hours in patients after OHCA [28, 29]. The guidelines also emphasise the need for more data on the optimal depth and length of cooling procedures after CA [27, 28]. The present study compares 48-hour therapy to 24-hour TTM therapy at 33 ± 1 °C. The standard length of TTM in Europe is 24 hours, and this duration was, therefore, selected as the standard care in the control group [30]. The reason that 48-hour duration was chosen for the intervention arm is based on various considerations. Prolonged cooling has been shown to be beneficial in neonates and newborns, where treatment duration is around 72 hours. A meta-analysis including 11 randomised trials showed a statistically significant reduction in the combined outcome of mortality or major neuro-developmental disability in patients until 18 months of age [31] compared to those maintained at normothermia. In most studies, cooling duration was 72 hours, and in two studies it was 48 hours. Thus, we considered it to be reasonable to prolong therapy for 48 hours, where a longer duration of cooling may be more effective but may have a higher likelihood of developing complications, such as infection. Recently, Kagawa and colleagues published a retrospective study on the duration of TTM, targeting a temperature of 33 °C. They compared patients who had been treated for shorter or longer than 28 hours and found no benefit but an increased risk of pneumonia and arrhythmia with longer treatment. However, this was a retrospective study, which makes it prone to bias since it is possible that patients with prolonged resuscitations were treated for longer periods of time [32].

Regarding inclusion and exclusion criteria, we decided to exclude patients for whom treatment could not be initiated within 240 min following arrest. The evidence regarding whether prompt or delayed TTM benefits patients is inconclusive, and it is impossible to evaluate in an observational trial since patients suffering more severe injuries may more easily show a decrease in temperature. As we did not know the optimal time window for beginning TTM in OHCA patients, we therefore set the relatively restrictive time limit of 240 min.

In addition, in the present trial we decided to exclude patients with a delay in the ROSC exceeding 60 min. The Hypothermia After Cardiac Arrest (HACA) trial focused on patients with a delay in the ROSC of less than 35 min, whereas the recent TTM trial by Nielsen and colleagues included patients with up to 170 min of cardiopulmonary resuscitation (CPR) [16, 33]. Some previous studies have indicated that TTM is more effective with shorter delays in the ROSC [34]. Thus, in the present study we decided to limit inclusion to patients with delays in the ROSC shorter than 60 min.

### The cooling methods

Similar to previous TTM trials [16], the TTH48 is planned as a pragmatic RCT. The participating centres can use either intravenous or superficial cooling methods according to local practices. It has been demonstrated that the use of invasive cooling devices results in a tighter temperature control compared to other tools [35], but neither of the two observational or the one randomised study comparing superficial versus intravenous cooling methods showed any effect on neurological outcome between the two techniques [36, 37]. Concerning the rewarming rate, various papers recommend a controlled increase in body temperature not exceeding 0.25 to 0.50 °C/h. Thus, we selected 0.5 °C/h, considering the lack of evidence on the optimal therapeutic approach for this issue. Importantly, a recent experimental study concluded that a rapid rewarming rate of 2.0 °C/h abolished all the beneficial effects of TTM, but this did not occur when the rewarming rate was set at 0.5–1.0 °C/h [38].

In conclusion, this is the first randomised clinical study conducted in adult OHCA patients comparing two different durations (24 and 48 hours) of TTM at 33 ± 1 °C. This study will provide information regarding whether this is associated with clinical benefit as well as relevant adverse events.

#### Trial status

Three hundred and four patients have been randomised by 1 February 2016 (Fig. 2). Allocation of the last of the 350 patients is anticipated to be late May 2016. Collection of outcome data will end late November 2016.

## Abbreviations

CA: cardiac arrest
CPC: Cerebral Performance Category score
CPR: cardiopulmonary resuscitation
DSMC: Data and Safety Monitoring Committee
e-CRF: web-based chart record form
EEG: electroencephalogram
EMR: electronic medical records
GCS: Glasgow Coma Score
ICU: intensive care unit
LLSTP: limitations on life-sustaining therapies procedure
MAP: mean arterial pressure
OHCA: out-of-hospital cardiac arrest
ROSC: return of spontaneous circulation
SAR: serious adverse reactions
SSEP: somatosensory evoked potentials
TH: therapeutic hypothermia
TTM: targeted temperature management
